# The effect of changes in cardiovascular activity on corneal biomechanics and pulsation in rabbits

**DOI:** 10.1038/s41598-020-79219-9

**Published:** 2020-12-17

**Authors:** Agnieszka Antończyk, Dominika Kubiak-Nowak, Wojciech Borawski, Zdzisław Kiełbowicz, Monika E. Danielewska

**Affiliations:** 1 Department of Surgery, Faculty of Veterinary Medicine, Wroclaw University of Environmental and Life Sciences, pl. Grunwaldzki 51, 50-366 Wrocław, Poland; 2 Department of Biomedical Engineering, Wroclaw University of Science and Technology, Wybrzeze Wyspianskiego 27, 50-370 Wrocław, Poland

**Keywords:** Circulation, Biological models, Medical research, Biomechanics, Cardiovascular biology

## Abstract

The aim was to assess the relationships between cardiovascular activity, corneal pulse characteristics, and corneal biomechanics in rabbits. Seventeen rabbits were randomly assigned to one of two anesthetic regimens to induce differences in arterial blood pressure and heart rate. Experimental protocol included measuring blood flow parameters in the ophthalmic artery by color Doppler imaging, corneal biomechanical parameters using a non-contact tonometer Corvis ST, and the corneal pulse (CP) signal using a non-contact ultrasonic technique. Statistically significantly lower mean values of normalized amplitudes of higher CP harmonics and changes in eight of the twelve corneal biomechanical parameters were observed in the rabbit group with lower arterial blood pressure and higher heart rate, intraocular pressure, and resistive index. The results of partial correlations showed that the CP signal energy and amplitude of its first harmonic correlate with the resistive index, diastolic and mean arterial pressures, whereas no statistically significant correlation was found between any of the CP parameters and intraocular pressure. Our pilot study indicates, for the first time, that non-contact and continuous measuring of corneal pulse allows indirectly assessing changes in cardiovascular activity when the confounding effect of intraocular pressure is eliminated.

## Introduction

With each heartbeat, blood pressure wave is propagated to the eye resulting in choroidal volume changes, pulsatile ocular tissue movements and intraocular pressure (IOP) variations. This phenomenon is known as the ocular pulse (OP)^1–4^. The OP signal characteristics have been found to be highly influenced by cardiovascular activity^2,5–9^. In particular, Trew et al.^9^ showed that ocular pulse volume, derived from the pulse amplitude and the duration of its waveform, decrease with increasing heart rate (HR). Further, the waveform of the corneal indentation pulse has been shown to correspond to the alteration in ocular blood supply related to several pathologies^10^. Correlation between the ocular pulse amplitude and the degree of carotid stenosis has been found in animal studies^4,11^. Recently, characteristic changes in a shape of corneal pulsation, named as an ocular dicrotic pulse, have been observed in healthy elderly subjects^6^ and glaucoma patients^7^. It is speculated that both vascular and biomechanical factors have contribution to these changes, however, explaining a mechanism of its formation without controlling those factors is difficult. Evaluating the relationship between cardiovascular parameters and corneal biomechanics and pulsation is difficult in in-vivo studies because it requires controlled levels of blood pressure and heart rate. To ease such an evaluation animal model are presented^12–14^.

Arterial blood pressure (ABP) and HR are cardiovascular variables that can be easily modified in an animal model. Several approaches, in which these parameters are modified, have been proposed to understand mechanisms of ocular physiology and pathology^12–14^. For example, Marcus et al. analyzed factors that determine the decrease of IOP in animal model triggering an increase of arterial blood pressure through acute exercise^14^. Another way of changing the ABP and HR is pharmacological induction. There are several medications influencing cardiovascular activity including, among others, sedative and anesthetic drugs. The specifics of examination of the anterior segment of the eye requires stable measurement conditions. In the case of an animal model, stable conditions are usually associated with sedation or general anesthesia. Hence, pharmacologically induced changes in heart activity with sedatives and/or anesthetics seem to be appropriate to evaluate the relationship between these changes and corneal pulsation and biomechanics. Furthermore, the advantage of this method is the ability to simultaneously perform hemodynamic and ocular measurements under controlled conditions.

The purpose of this study, in which control levels of cardiovascular activity is achieved pharmacologically, is to investigate the relationships between blood pressure, heart rate, corneal pulse characteristics, and corneal biomechanics in rabbits.

## Methods

### Animals

The study protocol for the experimental use of the animals was approved by the Animal Care and Ethics Committee of the Wroclaw University of Environmental and Life Sciences (No. 82/2017) and adhered to the ARRIVE guidelines and the EU Directive 2010/63/EU for animal experiments. Seventeen White New Zealand rabbits, aged 6 months and weighing from 2.9 to 3.8 kg without sex restrictions were used in this study. The rabbits were acclimatized for 2 weeks after shipment. Animals were kept individually in a stainless-steel cages under standard environmental conditions (15 to 20 °C, 40% to 60% humidity, 12 h in light conditions and 12 h in dark conditions). The rabbits were fed a commercial diet (Labofeed KH Standard) and had ad libitum access to water via 1-L water bottles with stainless steel ball-bearing sipper tubes (bottles were changed daily). Food and water were not withdrawn before treatment.

### Study design and animals preparation

The animals were randomly assigned one of two different anesthetic regimens: Group I ($n = 8$) and Group II ($n = 9$). The combinations of drugs used for premedication and general anesthesia allowed to induce differences in ABP and HR between groups. The choice of acepromazine and izoflurane combination was dictated by their vasodilator properties leading to lowering ABP^15,16^, whereas ketamine was used as only anesthetic agent that leads to an increase blood pressure and heart rate by activation of sympathetic nervous system^17^.

In Group I, rabbits received 0.5 mg/kg acepromazine (Calmivet, Vétoquinol, Luré, France) and 0.5 mg/kg midazolam (Midanium, Polfa Warszawa, Poland) intramuscularly as a premedication. To induce general anesthesia propofol (Propofol-Lipuro 1%, B. Braun Melsungen AG, Germany) at initial dose of 0.5 mg/kg was injected. After local anesthesia of larynx with diluted 1:1 2% lidocaine (Lignocainum Hydrochloricum WZF 2%, Polfa Warszawa, Poland) rabbits were intubated with uncuffed endotracheal tube (internal diameter 3.0–3.5 mm) and general anesthesia was maintained with oxygen-volatilized isoflurane (1.5–2 vol.%, IsoVet, Piramal Healthcare United Kingdom).

In Group II, rabbits were given medetomidine (0.3 mg/kg, Cepetor, CP-Pharma Handelsges, Germany) and ketamine (20 mg/kg, Bioketan, Vetoquinol Biowet, Poland) intramuscularly. Animals were intubated with uncuffed endotracheal tube (internal diameter 3.0–3.5 mm) and received pure oxygen. Rabbits from both groups were breathing spontaneously.

After obtaining surgical plane of anesthesia the left ear of each rabbit was clipped and the skin cleaned with alcohol, a 22 G catheter was placed in the marginal ear vein for anesthetics and fluid injections. Right ear was used to central auricular artery catheterization (20 G catheter). Catheter was connected via saline-filled non-compliant tubing (regularly flushed) to a calibrated pressure transducer (zeroed level with the thoracic inlet) to obtain direct measurement of the heart rate, systolic, diastolic and mean arterial blood pressure (HR, SBP, DBP, and MBP, respectively), (Datex-Ohmeda GE Healthcare S5, Finland). Arterial oxygen saturation (SpO_2_) was monitored using a pulse oximeter with a probe placed on the tongue (Datex-Ohmeda GE Healthcare S5, Finland). Sidestream capnometry was used to measure end tidal carbon dioxide and respiratory rate (Datex-Ohmeda GE Healthcare S5, Finland). Since full animals’ instrumentation to the end of all measurements, all the above parameters were monitored every 5 min and recorded every 20 min. Heating pad was used to maintain normal body temperature.

The experimental protocol followed the sequence: (1) color Doppler imaging of the external ophthalmic artery, (2) measurement of the corneal biomechanical parameters, (3) measurement of the corneal pulse (CP) signal.

### Color Doppler imaging (CDI)

Blood flow measurement was performed in both groups using an ultrasound system (ESAOTE MyLab ClassC) equipped with a 4–13 MHz linear ultrasound transducer. After achieving anesthesia plan, a layer of sterile aqueous gel was applied to the corneal surface of left eye, and the transducer was gently positioned with minimal pressure in the horizontal plane. The long axis of the transducer was held horizontally between the lateral and medial canthus with the marker pointing nasally. The external ophthalmic artery was identified near the optic nerve by color Doppler imaging. The peak systolic velocity (PSV) and end diastolic velocity (EDV) were measured and mean values of PSV and EDV from three pulse waveforms were calculated. Arterial resistive index (RI) of the blood flow was calculated using the formula $\text {RI} = (\text {PSV}-\text {EDV}) / \text {PSV}$.

### Corneal biomechanics measurement

Following the external ophthalmic artery imaging, measurements of the corneal biomechanical parameters of the left eye were acquired in both groups using a non-contact tonometer Corvis ST (OCULUS Optikgeräte GmbH, Wetzlar, Germany). Initially, the rabbit’s head, held by an anesthesiologist, was positioned such that the cornea surface was facing the tonometer horizontally. This guaranteed successful records of deformation profiles of the cornea under the effect of air pressure with a high-speed Scheimpflug camera and provided 12 parameters stemming from the imaging analysis. Those parameters included: uncorrected intraocular pressure (IOP), central corneal thickness (CCT), the time from the start until the first and second applanation (A1T and A2T, respectively), the length of the flattened cornea during the first and second applanation (A1L and A2L), the corneal velocity at the two applanation events (A1V and A2V), the time from the start till the highest concavity of cornea (HCT), the maximum deformation amplitude (DA), the distance between the corneal bending points (PD), and the radius of the curvature at the corneal apex (R) at the time of the highest concavity. The measurement was repeated three times for each eye at inter-measurement intervals of at least 2 min.

### Measuring the corneal pulse signal

After the corneal biomechanics measurements, when the animal stayed laid, the pediatric size wire speculum was used in the left eye, in a manner preventing direct pressure on the cornea and the eye globe, to retract the eyelids and nictitating membrane, as well as to enhance the exposure of the conjunctiva, cornea, and eye globe during the measurements of the corneal pulse (CP) signal. Then, the CP signal was registered in both groups using a non-contact ultrasonic technique (UltraLab, Wrocław, Poland)^18^. An ultrasonic transducer was mounted in a specially constructed holder and placed in front of the central cornea within its working distance (around 12–15 mm), as performed in our recent animal study^19^. Five 30-s CP signals were measured for each eye with the sampling frequency of 400 Hz. Due to the ocular surface expansion^1^, the CP signal measured at its central area was chosen as a representative signal of the ocular pulse phenomenon.

### Signal analysis

To compute time and spectral parameters of the CP signal, the raw signal data were preprocessed numerically using a custom-made algorithm in MATLAB (MathWorks, Inc., Natick, MA, USA). This procedure included linear detrending and filtering in the range from 1.3 to 30 Hz. This range was similar to that from our earlier work^19^. However, the lower bound was now adjusted to the lower mean of the heart rate frequency in Group II. After visual inspection of the signals performed by a signal-processing expert, three 30-s artefact-free CP signals were chosen from each rabbit for further analysis. In the time domain, the CP signal energy was computed as a root mean squared (RMS) value, whereas in the frequency domain, amplitudes of the first four harmonics of the CP signal (A1, A2, A3, and, A4) were calculated using the Fourier transform. The first CP harmonic corresponds to the frequency of the rabbit’s heart rate. The amplitudes of the higher harmonics were normalized to that of the first one, resulting in $\text {A2n} = \text {A2}/\text {A1}$, $\text {A3n} = \text {A3}/\text {A1}$, $\text {A4n} = \text {A4}/\text {A1}$. Finally, all CP parameters were averaged for each rabbit.

All experimental data are included in the Supplementary Data file (.xlsx) of Supplementary Information.

### Statistical analysis

Statistical analysis included standard descriptive statistics and normality testing using the Kolmogorov–Smirnov test with the Lilliefors correction. The null hypothesis of normality was not rejected for any of the considered parameters in both groups of rabbits ($p > 0.05$). Furthermore, verification of the null hypothesis about the equality of variances with the Levene’s test showed that for the five parameters the variances were not equal ($p < 0.05$). Therefore, to test the differences between the means of the two groups the unpaired *t*-test was used either with the assumption of unequal variances (in the case of the aforementioned five parameters) or equal variances (for the remaining parameters). To compute the mutual relationship between any two parameters, partial correlation was performed, with Group set as a control variable. Additionally, we have performed partial correlation analysis with Group and IOP set as control variables to compute those relationships, when the confounding factor of IOP was also removed. The significance level $\alpha$ was set to 0.05 in all the abovementioned tests.

When testing for zero correlation, the sample size of 17 used in this study would deemed significant correlation values those that are larger than 0.6 (or less than $-0.6$)^20^. Nevertheless, in this study we also consider absolute correlation values close to 0.6 for which the test power will be just below 80% for considering them significant.

Temporal changes of the mean values of HR, SBP, DBP, and MBP within each group at different time points (0 mins, 20 mins, and 40 mins) were tested using the one-way repeated measures analysis of variance (ANOVA) with Bonferroni corrected $\alpha$ = 0.017.

Calculations were performed using SPSS 22.0 (SPSS, Inc., Chicago, IL, USA).

## Results

**Figure 1:**
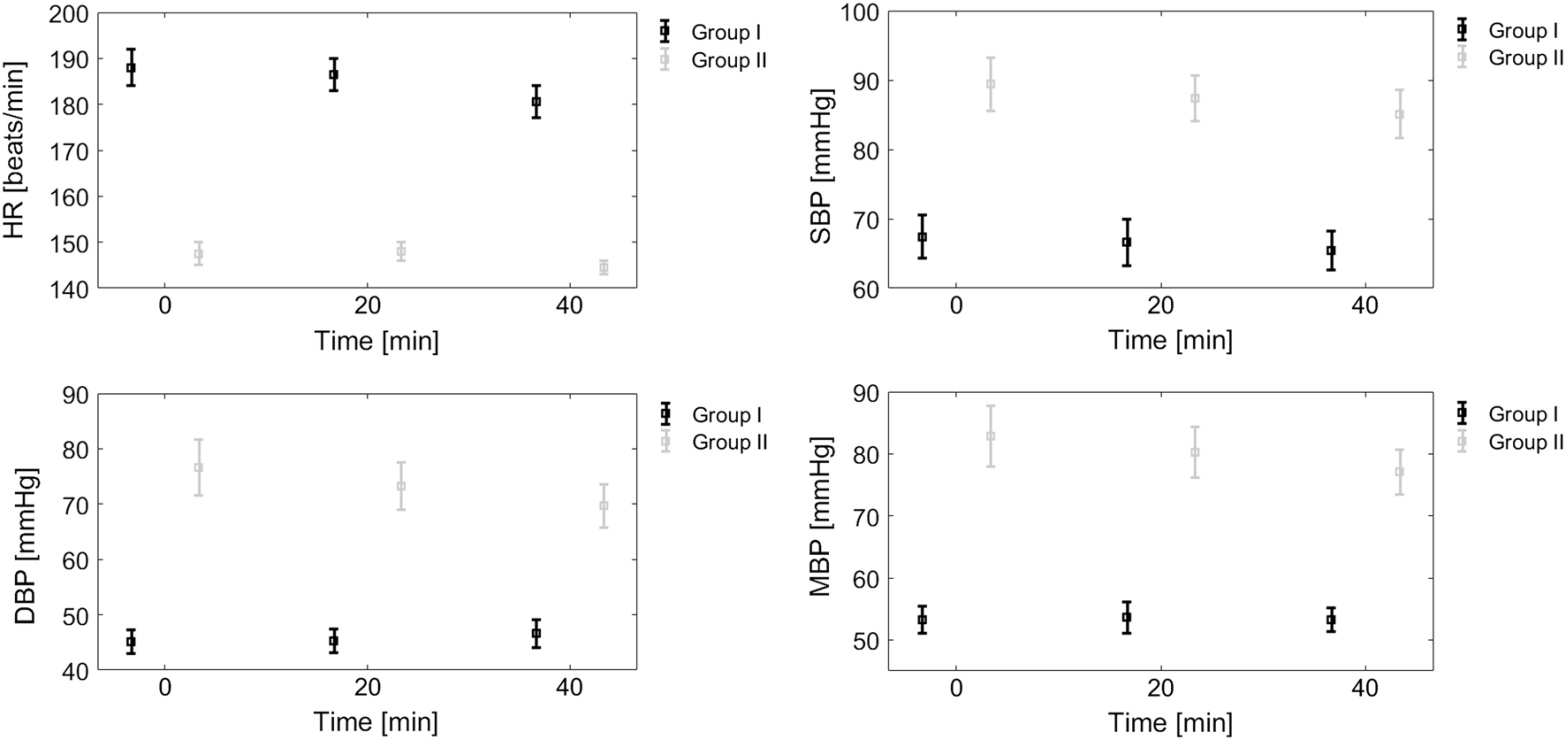
Group averages of heart rate (HR), systolic (SBP), diastolic (DBP) and mean arterial blood pressure (MBP) at different time points in Group I and II. Error bars denote one standard error.

Time from induction of general anesthesia to the end of all measurements did not exceed 40 min in both groups. Hemodynamic parameters were stable throughout the experiment (Fig. 1). In Group I, there were no intra-group variations in terms of HR, SBP, DBP, and MBP. In Group II, only DBP decreased statistically significantly from 72.5 mmHg at 20 min to 68.7 mmHg at 40 min ($p = 0.002$).

Mean values, standard deviations and ranges of all considered parameters together with the results of Levene’s test and unpaired *t*-test are given in Table 1.

**Table 1:** Descriptive statistics for all considered parameters and the results of Levene’s test and unpaired *t*-test.

Parameter	Mean ± SD (ranges)		Levene’s test		*t*-test	
	Group I	Group II	*F*	*p*-value	*t*	*p*-value
HR (beats/min)	224 ± 20	150 ± 12	2.910	0.109	9.615	$<\mathbf{0} .\mathbf{001}$
	(200–250)	(126–171)				
SBP (mmHg)	67 ± 6	88 ± 10	2.353	0.146	5.223	$< \mathbf{0} .\mathbf{001}$
	(59–77)	(71–99)				
DBP (mmHg)	46 ± 6	70 ± 10	1.052	0.321	6.434	$<\mathbf{0} .\mathbf{001}$
	(39–53)	(55–88)				
MBP (mmHg)	54 ± 5	78 ± 10	2.252	0.154	6.758	$<\mathbf{0} .\mathbf{001}$
	(48–60)	(62–93)				
PSV (cm/s)	25.0 ± 7.5	22.9 ± 9.8	0.315	0.583	0.500	0.624
	(14.6–33.8)	(11.9–38.8)				
EDV (cm/s)	10.1 ± 3.9	12.2 ± 7.0	2.306	0.150	0.739	0.472
	(4.9–17.0)	(4.6–26.4)				
RI (–)	0.60 ± 0.08	0.48 ± 0.10	0.230	0.638	2.593	**0**.**020**
	(0.50–0.73)	(0.32–0.64)				
IOP (mmHg)	14.7 ± 2.4	9.7 ± 2.1	0.001	0.974	4.654	$<\mathbf{0} .\mathbf{001}$
	(10.3–17.7)	(6.5–12.0)				
CCT ($\upmu$m)	371 ± 34	361 ± 25	0.404	0.534	0.726	0.479
	(333–438)	(322–401)				
A1T (ms)	7.0 ± 0.4	6.7 ± 0.2	2.570	0.130	2.692	**0**.**017**
	(6.5–7.5)	(6.4–6.9)				
A2T (ms)	22.5 ± 3.4	21.8 ± 0.3	4.866	0.043	0.626	0.551
	(21.0–30.8)	(21.3–22.3)				
A1L (mm)	2.0 ± 0.3	1.8 ± 0.08	11.852	0.004	2.547	**0**.**033**
	(1.8–2.4)	(1.7–2.0)				
A2L (mm)	1.6 ± 0.4	1.2 ± 0.4	0.315	0.583	2.139	**0**.**049**
	(1.1–2.1)	(0.7–1.9)				
A1V (m/s)	0.14 ± 0.02	0.17 ± 0.03	1.283	0.275	2.483	**0**.**025**
	(0.12–0.17)	(0.14–0.23)				
A2V (m/s)	$-0.37$ ± 0.20	$-0.83$ ± 0.17	$< 0.001$	0.990	5.274	$<\mathbf{0} .\mathbf{001}$
	($$-0.71$$to 0)	($$-1.05$$ to $$-0.55)$$				
HCT (ms)	16.9 ± 0.4	16.9 ± 0.6	1.789	0.201	0.186	0.855
	(16.5–17.6)	(15.9–17.6)				
DA (mm)	1.2 ± 0.1	1.4 ± 0.2	1.713	0.210	4.268	**0**.**001**
	(1.0–1.3)	(1.2–1.6)				
PD (mm)	4.2 ± 1.3	3.2 ± 0.8	5.323	0.036	1.913	0.082
	(2.2–5.2)	(2.5–4.4)				
R (mm)	5.0 ± 0.4	4.5 ± 0.3	1.952	0.183	3.098	**0**.**007**
	(4.4–5.7)	(4.1–5.0)				
RMS ($\upmu$m)	13.1 ± 4.6	14.0 ± 6.9	1.312	0.270	0.316	0.756
	(6.2–17.4)	(5.5–25.9)				
A1 ($\upmu$m)	7.2 ± 3.1	7.4 ± 4.3	0.978	0.338	0.085	0.933
	(2.4–10.2)	(2.6–14.1)				
A2n (–)	0.36 ± 0.11	0.19 ± 0.06	3.771	0.071	4.522	$<\mathbf{0} .\mathbf{001}$
	(0.27–0.53)	(0.13–0.28)				
A3n (–)	0.15 ± 0.06	0.06 ± 0.03	4.784	0.045	4.120	**0**.**002**
	(0.07–0.24)	(0.03–0.12)				
A4n (–)	0.11 ± 0.05	0.04 ± 0.02	5.037	0.040	4.030	**0**.**003**
	(0.06–0.18)	(0.01–0.07)				

Bolded values indicate $p < 0.05$ in the *t*-test.

**Figure 2:**
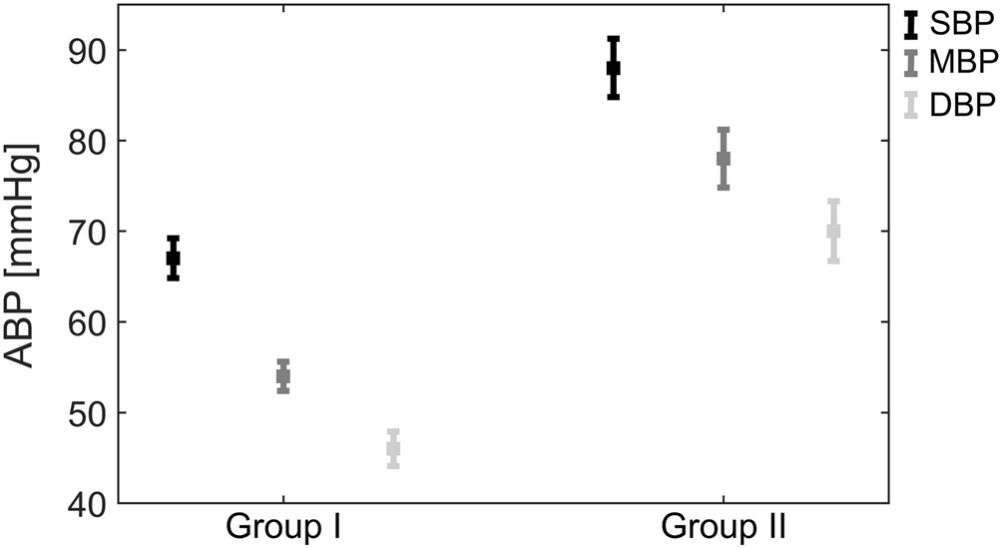
Means ± standard errors of blood pressures for the two study groups. SBP—systolic blood pressure, MBP—mean blood pressure, DBP—diastolic blood pressure.

**Figure 3:**
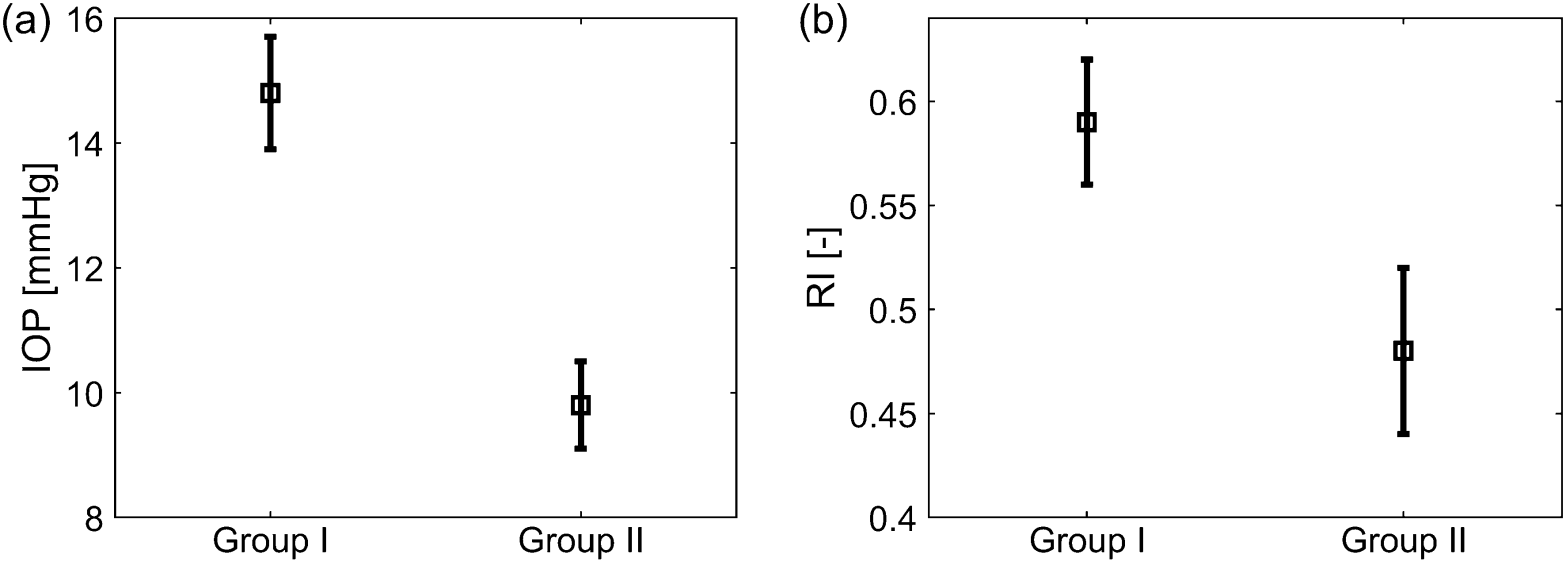
Means ± standard errors of uncorrected intraocular pressure, IOP, (**a**) and arterial resistive index, RI, (**b**) for the two groups.

Group II is characterized by statistically significantly higher values of SBP, DBP, and MBP ($p < 0.001$) than Group I and lower values of HR ($p < 0.001$), IOP ($p < 0.001$) and RI ($p = 0.020$). The differences for blood pressures are shown in Fig. 2, whereas for IOP and RI are presented in Fig. 3.

**Figure 4:**
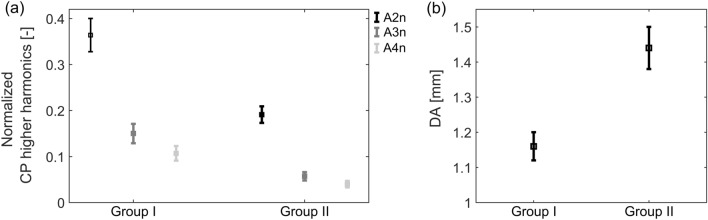
Means ± standard errors of the normalized amplitudes of the second, third, and fourth harmonic (A2n, A3n, A4n) of the corneal pulse (CP) signal (**a**) and the maximum deformation amplitude (DA) (**b**) for the two study groups.

Significant differences between the two groups in the three considered arterial blood pressures and HR did not implicate such a difference in the energy of the CP signal ($p = 0.756$ for RMS and $p = 0.933$ for A1). However, they resulted in significant changes of the spectral content of the CP signal in both groups, revealing lower mean values of A2n, A3n, and A4n in Group II (see Table 1 and Fig. 4a).

There were statistically significant differences between the two groups for eight of the 12 considered corneal biomechanical parameters, the four remaining ones being CCT, PD, A2T, and HCT (see Table 1). Figure 4b shows mean values of selected corneal biomechanical parameter, the maximum deformation amplitude (DA). The results of partial correlations, with Group set as a control variable and with Group and IOP set as control variables, are presented in Tables 2 and 3, respectively. Two of the CP parameters, RMS and A1, were found to positively correlate with RI, DBP, and MBP. The correlations between RMS, A1 and DBP or MBP became stronger when the IOP was eliminated additionally to the Group as a potential confounding factor in the study. On the other hand, the normalized amplitude of the second CP harmonic was found to correlate with biomechanical parameters of the cornea, such as: A2T, and A1L. This result remains at a similar level of statistical significance in both considered types of partial correlation analyses.

**Table 2:** Results of partial correlation analysis (cor) between the CP signal parameters and those of blood and intraocular pressure, blood flow in the external ophthalmic artery, and corneal biomechanics, with Group used as a control variable.

Parameters	cor	HR	SBP	DBP	MBP	PSV	EDV	RI	IOP	CCT	A1T	A2T	A1L	A2L	A1V	A2V	HCT	DA	PD	R
RMS	*R*	$-.108$	$-.298$	$-\mathbf .623$	$-\mathbf .550$	$-.131$	$-.361$	**.635**	$-.293$	$-.436$	$-.226$	$-.241$	$-.174$	.160	.174	$-.440$	.376	.488	$-.227$	$-.284$
	*p*	.691	.262	**.010**	**.027**	.628	.170	**.008**	.271	.092	.400	.368	.518	.555	.520	.088	.151	.055	.397	.287
A1	*R*	-.095	$-.279$	$-\mathbf .587$	$-\mathbf .526$	$-.174$	$-.380$	**.601**	$-.325$	$-.361$	$-.270$	$-.198$	$-.158$	.128	.210	$-.415$	.370	.449	$-.172$	$-.242$
	*p*	.727	.296	**.017**	**.036**	.519	.146	**.014**	.220	.170	.312	.462	.559	.635	.435	.110	.158	.081	.524	.366
A2n	*R*	$$-.027$$	$$-.100$$	.036	$$-.074$$	.010	$$-.013 $$	.094	.199	.467	.187	** .587**	**.542**	$$-.330$$	$$-.096$$	.288	.427	$$-.063$$	.160	$$-.207$$
	*p*	.921	.712	.896	.786	.970	.963	.730	.460	.068	.487	**.017**	**.030**	.213	.724	.279	.099	.818	.553	.441
A3n	*R*	.294	$$-.105$$	.108	.017	.299	.198	.054	$$-.025 $$	.402	$$-.142$$	.076	.347	$$-.050$$	$$-.046$$	.081	$$-.011$$	.068	.341	$$-.161$$
	*p*	.269	.697	.692	.949	.261	.463	.844	.927	.123	.601	.781	.188	.854	.866	.764	.968	.803	.196	.551
A4n	*R*	$-.072$	-.101	.104	.020	.252	.268	$-.082$	.130	.082	.178	.115	.251	$-.179$	$-.094$	.057	$-.024$	$-.102$	.030	$-.204$
	*p*	.792	.709	.703	.940	.346	.316	.763	.631	.762	.511	.671	.348	.507	.728	.835	.929	.708	.913	.448

Statistically significant results are bolded.

**Table 3:** Results of partial correlation analysis (cor) between the CP signal parameters and those of blood and intraocular pressure, blood flow in the external ophthalmic artery, and corneal biomechanics, with Group and IOP used as control variables.

Parameters	cor	HR	SBP	DBP	MBP	PSV	EDV	RI	CCT	A1T	A2T	A1L	A2L	A1V	A2V	HCT	DA	PD	R
RMS	*R*	$-.128$	$-.234$	$-\mathbf .671$	$-\mathbf .563$	$-.157$	$-.362$	**.594**	$-.465$	.092	$-.246$	$-.186$	.244	-.137	-.356	.472	.416	-.302	$-.289$
	*p*	.651	.401	**.006**	**.029**	.575	.185	**.020**	.081	.743	.378	.506	.380	.627	.193	.075	.123	.274	.295
A1	*R*	-.117	$-.205$	$-\mathbf .643$	$-\mathbf .542$	$-.206$	$-.385$	**.549**	$-.392$	.056	$-.202$	$-.172$	.221	-.120	$-.311$	.479	.328	$-.252$	$-.248$
	*p*	.679	.463	**.010**	**.037**	.460	.157	**.034**	.149	.842	.470	.541	.429	.671	.260	.071	.233	.364	.373
A2n	*R*	$$-.018$$	$$-.167$$	.049	$$-.084$$	.024	$$-.023 $$	.179	.483	.019	** .595**	**.556**	$$-.394$$	.132	.223	.401	.113	.206	$$-.217$$
	*p*	.950	.551	.864	.766	.934	.934	.524	.068	.948	**.019**	**.032**	.147	.640	.423	.138	.688	.461	.438
A3n	*R*	.293	$-.103$	.106	.019	.298	.199	.048	.401	$-.276$	.076	.347	$-.046$	$-.122$	.107	$-.005$	.071	.343	$-.160$
	*p*	.289	.716	.706	.948	.281	.477	.866	.138	.320	.787	.206	.872	.665	.704	.985	.802	.211	.568
A4n	*R*	$-.066$	-.146	.113	.015	.264	.264	$-.039$	.087	.140	.113	.255	$-.217$	.027	-.008	-.054	$-.014$	.056	$-.209$
	*p*	.815	.604	.690	.958	.342	.342	.891	.758	.618	.688	.359	.438	.924	.978	.847	.962	.842	.454

Statistically significant results are bolded.

**Table 4:** Data comparison between published studies on relationship between arterial blood pressure (ABP) and intraocular pressure (IOP).

References	Treatment	Species	*N*	Relationship between ABP and IOP
^21^	No	Human	4764	Positive
^22^	No	Human	4926	Positive
^13^	Norepinephrine (0.03 to 30 $\upmu$g/kg) and angiotensin II (0.03 to 1.0 $\upmu$g/kg)	Cat	32	Positive
	Methacholine (0.01 to 3.0 $\upmu$g/kg)	Cat	40	Positive
	Isoproterenol (0.03 to 1.0 $\upmu$g/kg)	Cat	40	Positive
^12^	No	Rat	39	Negative in hypertensive rats; no in normotensive rats
	Kallikrein (150 KU/kg)	Rat	14	Positive
	Bradykinin (3 $\upmu$g/kg)	Rat	26	No
	Angiotensin I (3 $\upmu$g/kg)	Rat	14	No
	Angiotensin II (3 $\upmu$g/kg)	Rat	27	No
	Bloodletting (2 mL)	Rat	14	No
^23^	Propofol (0.98 mg/kg)	Human	20	Positive
^24^	Fentanyl (0.01 mg/kg)	Dog	10	No
	Medetomidine (0.01 mg/kg)	Dog	10	No
	Acepromazine (0.02 mg/kg)	Dog	10	No
	Butorphanol (0.2 mg/kg)	Dog	10	No
^25^	Thiopental sodium (5 to 6 mg/kg)	Human	23	Positive
	Remifentanil (CRI)			
	Desflurane (inhalant)			
	Rocuronium (1 mg/kg)			
	Propofol (1.5 to 2.5 mg/kg bolus) + CRI remifentanil (CRI) rocuronium (1 mg/kg)	Human	23	Positive

## Discussion

Alterations in cardiovascular activity is of interest in studying many systemic and ocular diseases^26,27^. The pathophysiology of cardiovascular diseases reflects in changes in eye dynamics^4^ and ocular rigidity^28^. The potential of detecting vascular changes using contactless techniques of the ocular pulse measurement has been reported in animal studies^4,11^. However, in-vivo non-contact assessment of changes in ocular tissues biomechanics based on natural tissues expansion due to the heart rate is still challenging. This animal study, in which the arterial blood pressure (ABP) and the heart rate (HR) was controlled pharmacologically, contributes to those findings and reveals the relationships between parameters of corneal pulsation and biomechanics and those of cardiovascular activity.

Acepromazine and isoflurane have been known to reduce ABP in human^16^ and animals^15^, whereas ketamine showed to exert opposite effect increasing blood pressure^15,17^. In this study, to induce differences in ABP and HR between two rabbit groups different anesthetic protocols were used with acepromazine and isoflurane in Group I and ketamine and medetomidine in Group II. Mean values of systolic, diastolic and mean arterial pressures were found to be statistically significantly lower in Group I than those in Group II. This was expected according to the previous results^15–17,29^. The purpose of the selected drugs was solely to differentiate the two groups in terms of cardiovascular parameters.

A close relationship between ABP and IOP has been reported in human^22,23^, dogs^24^, cats^13^, rats^12^ and rabbits^21^. However, different methodologies used in the studies of other authors may resulted in different correlation between ABP and IOP (see Table 4). Gherezghiher et al. has concluded that IOP is largely influenced by ABP changes in the acute phase of drug action^13^. Furthermore, the IOP response to drugs is the algebraic sum of changes in the mean systemic pressure and the direct ocular effects of agents used. Therefore, the interpretation of the relationship between ABP and IOP should consider the drugs used, their combinations, dose, and route of administration.

In this study, IOP has reached statistically significantly higher values in rabbits with lower ABP (Group I) than in rabbits with higher ABP (Group II). These findings can be explained by the employed anesthetic protocols. The drugs have been selected to emphasize the differences in blood pressure and heart rate parameters between the two groups. However, apart from the desired events, drugs directly affect IOP and exert additional effects like myorelaxation or aqueous humor production and outflow which also affect IOP.

Assuming an average value of natural IOP in rabbits in the range of 15–23 mmHg^14^, it can be claimed that after anesthesia, the mean IOP in Group I might not have changed or slightly decreased, whereas in Group II it might have significantly decreased. Volatile anaesthetics, like isoflurane used in Group I, either slightly reduce or have no effect on IOP^30,31^. Moreover, additionally used midazolam and acepromazine has been shown to have no significant effect on IOP^30^. On the other hand, it has been reported that medetomidine, used in Group II, reduces IOP^32^, as well as combination of alfa-2 agonist and ketamine has the same effect on IOP^33^.

Three possible mechanisms behind the reduction in IOP mediated by a2-adrenoceptor agonists have been previously suggested: (1) a reduction in aqueous humor production caused by the activation of pre-junctional a2-adrenoceptors, (2) vasoconstriction in the ciliary body triggered by activation of post-junctional a2-adrenoceptors, and (3) inhibition of adenylyl cyclase by the activation of epithelial a2-adrenoceptors to suppress the production of aqueous humor^34^. Furthermore, the intravenous administration of medetomidine has been shown to cause miosis in dogs^35^. Miosis is known to reduce IOP by increasing the outflow of aqueous humour^36^, hence, pupil size is an another important factor that can affect IOP. Additionally, medetomidine effects are decreasing the central venous pressure and miorelaxation, including relaxation of extraocular and retrobulbar muscles. These effects can also contribute to reduction of IOP. In addition, an influence of ketamine on the IOP values in Group II should be also considered. Some reports suggested that ketamine elevates IOP^37^, whereas others that there is no effect^30^. The latter are further supported by animal studies, which have shown that IOP tends to be stable after ketamine administration^30^. Based on aforementioned findings and assuming a negligible influence of volatile anesthetics (Group I) and ketamine (Group II) on the IOP, it can be concluded that observed lower mean value of IOP in Group II results mainly from the use of medetomidine.

Mean arterial pressure, ocular circulation, specifically pressure within the choroid, and absolute IOP are the main determinants of the ocular pressure-volume relationship^38–40^. Changes in intraocular volume, caused by pulsatile arterial inflow and steady venous outflow^41^, are accompanied by changes in parameters and signals of the ocular pulse, ocular pulse amplitude^40^, pulsatile ocular blood flow^38,42^, fundus pulse amplitude^3,43^, cornea, sclera and retina movements^1–3,44^. Ocular rigidity, expressing elastic properties of the eye coat (cornea plus sclera), influences all abovementioned characteristics^45^. In our study, the corneal pulse (CP) signal was measured as a representative signal of the ocular pulse. The applied non-contact ultrasonic technique enabled to register the CP signal as a natural pulsatile corneal expansion without disturbing its natural biomechanical properties. Differences in ABP and HR in both considered rabbit groups did not implicate corresponding differences in the energy of the CP signals (stable RMS values), but implicate differences in their spectral content. Specifically, significantly higher mean amplitudes of the second (A2n), third (A3n), and fourth (A4n) frequency harmonics of the CP signals were observed in Group I characterized by higher IOP and HR, and lower SBP, DBP, and MBP, than those in Group II.

Earlier studies showed that the elevation in IOP increases ocular rigidity, expressed in higher mechanical resistance of ocular walls^45,46^, and it was also reflected in spectral changes of the CP signals in glaucoma patients after the period of washout from antiglaucoma medications^47^. Taking the above findings into account, we conclude that in this study rabbits in Group I have higher ocular rigidity than that in Group II. Also, higher ocular stiffness can lead to lower internal damping, which, in turn, reflects in higher CP harmonic vibrations as a corneal response to pulsatile component of IOP. The results of corneal biomechanical properties, additionally measured with Corvis ST, are consistent with aforementioned mechanism. In particular, in Group I statistically significantly higher value of the first applanation length (A1L) and lower value of deformation amplitude (DA) were obtained, where both parameters correspond to higher IOP level in this group in comparison to those achieved in Group II. It has been shown that DA depends strongly on IOP^46^, whereas A1L is mostly affected by central corneal thickness and age of the subjects rather by a change in IOP. Therefore, A1L can be used to describe corneal stiffness. In this study, partial correlation analysis revealed that the normalized amplitude of the second CP harmonic, A2n, statistically significantly positively correlate with A1L, even when the IOP was removed as a confounding factor. So, our findings clearly demonstrate that the second harmonic of the CP signal could be utilized for estimating measures of corneal stiffness in an indirect manner. Further, according to our previous study with glaucoma patients who underwent canaloplasty combined with phacoemulsification^47^, changes in the amplitude of the third CP harmonic reflected alterations in corneoscleral stiffness caused by the surgery. Hence, the proposed analysis of the higher harmonics of the CP signal might be useful in the future to indirectly predict biomechanical alterations that appear in compromised corneas, such as those affected by keratoconus^48,49^, refractive surgery^50^, or diabetes^51^, and separate them from the changes in cardiovascular activity expressed mainly in the first harmonic of the CP signal corresponding to the heart rate.

Furthermore, difference in ABP and IOP values obtained between both rabbit groups resulted also in different values of the resistive index (RI) in ophthalmic artery as assessed by color Doppler imaging. The RI is considered to reflect vascular resistance peripheral to the measuring location^52^ dependent also on the degree of arterial stiffness. In our study, we found lower RI in Group II, when compared to Group I. These results are in agreement with a number of previous experiments which have shown that despite the ketamine raising the arterial blood pressure, RI is reduced^53–55^. The RI reduction was observed after combination of ketamine/alpha-2 agonist administration in large arteries in rabbits^53^. Similar effect of ketamine in smaller ocular vessels was obtained in cats^55^ and dogs^54^. This effect was supposed to be caused by direct acting of ketamine on the vascular smooth muscle layer causing its relaxation by decreasing $\text {Ca}^{2+}$ influx and by inhibiting mobilization of intracellular $\text {Ca}^{2+}$. Reicher *et al*.^56^ measured a marked cerebral vasodilatation in rabbits using ketamine alone. This effect was associated with ketamine-mediated inhibition of tension-dependent $\text {Ca}^{2+}$ influx in myocytes of the vessel wall^57^. Furthermore, in this study, based on partial correlation analysis, RI was found to correlate significantly only with two of the CP parameters—energy computed as a root mean squared (RMS) value and amplitude of the first harmonic (A1) related to the heart rate. With increasing RI the RMS and A1 increase, whereas there were negative correlations between RMS and DBP or MBP, as well as between A1 and DBP or MBP. Hence, RMS and A1 may indirectly reflect ocular volumetric changes and they are shown to depend on retrobulbar ocular hemodynamic parameter (RI). Several animal and human studies have shown that ocular volume changes, in the form of ocular pulse amplitude is directly related to the rigidity of the ocular coat^39,45^. Higher ocular pulse amplitude has been found in eyes with increased ocular rigidity, corresponding to a higher rigidity coefficient or a higher IOP level^45^ and in eyes with increased RI in retrobulbar vessels^58^. Here, significant association between RI and RMS or A1 parameters is consistent with aforementioned studies, underlying the role the CP signal characteristic may have in estimating variations in ocular volume related to changes in vascular resistance and cardiovascular activity, when the IOP was eliminated as a confounding factor. Hence, the proposed analysis of the CP signal measured using a non-contact technique could offer in the future additional supportive parameters to standard cardiovascular parameters having potential physiological and clinical utility in systemic diseases. However, further research including a larger number of patients with systemic diseases should be performed to form any guidelines for clinical care.

Limitations of this study are the small number of animals and lack of possibility to compare relative values of ocular and cardiovascular parameters before and after anesthesia within the groups. Thus, there is a need to confirm the obtained results in a larger study population. In that sense, our study has a pilot character. All measurements of IOP should not be treated in their absolute terms, because no calibration curve is available for quantifying IOP measurements in rabbits using Corvis ST. Further, results obtained with RI should be interpreted with caution, because the CDI technique does not reflect volumetric blood flow (no information about vessel diameter is available), but blood flow velocity^59^. Some concerns have been raised on RI as a good measure for peripheral vascular resistance. In the case of central retinal artery, RI was found not to be a valid indicator of vascular resistance^52^. Notice that RI was found to alter not by vascular resistance alone but by the combination of vascular resistance and vascular compliance^60^.

Since arterial blood pressure is inherently related to intraocular pressure, it may be difficult to disentangle the influence of the two pressures on changes in the CP signal. However, in this *in-vivo* animal experiment, for the first time, results of partial correlations clearly demonstrate that the RMS and A1 of the CP signal correlate with the resistive index, diastolic and mean arterial pressures, whereas no statistically significant correlation was found between any of the CP parameters and intraocular pressure. The main conclusion of this study is that changes in the energy and amplitude of the first harmonic of the CP signal show stronger dependence on the parameters of arterial blood pressure than intraocular pressure. This study indicates that by using the contactless and continuous method for measuring corneal pulse, one could indirectly assess changes in cardiovascular activity regardless of intraocular pressure.The proposed methodology might be helpful in exploring the significance of characteristics of the eye dynamics in the diagnosis of cardiovascular diseases.

## Supplementary Information

Supplementary Information.

## Data Availability

All data generated or analysed during this study are included in this published article (and its Supplementary Information files).
