# Author Correction: The effect of changes in cardiovascular activity on corneal biomechanics and pulsation in rabbits

**DOI:** 10.1038/s41598-021-88637-2

**Published:** 2021-04-21

**Authors:** Agnieszka Antończyk, Dominika Kubiak-Nowak, Wojciech Borawski, Zdzisław Kiełbowicz, Monika E. Danielewska

**Affiliations:** 1 Department of Surgery, Faculty of Veterinary Medicine, Wroclaw University of Environmental and Life Sciences, pl. Grunwaldzki 51, 50‑366 Wrocław, Poland; 2 Department of Biomedical Engineering, Wroclaw University of Science and Technology, Wybrzeze Wyspianskiego 27, 50‑370 Wrocław, Poland

Correction to: *Scientific Reports* 10.1038/s41598-020-79219-9, published online 17 December 2020

The Funding section in this Article was omitted. The Funding section should read:

“The research is financed under the Leading Research Groups support project from the subsidy increased for the period 2020–2025 in the amount of 2% of the subsidy referred to Art. 387 (3) of the Law of 20 July 2018 on Higher Education and Science, obtained in 2019.”

